# Enlarged striatal volume in adults with ADHD carrying the 9-6 haplotype of the dopamine transporter gene *DAT1*

**DOI:** 10.1007/s00702-016-1521-x

**Published:** 2016-03-02

**Authors:** A. Marten H. Onnink, Barbara Franke, Kimm van Hulzen, Marcel P. Zwiers, Jeanette C. Mostert, Aart H. Schene, Dirk J. Heslenfeld, Jaap Oosterlaan, Pieter J. Hoekstra, Catharina A. Hartman, Alejandro Arias Vasquez, Cornelis C. Kan, Jan Buitelaar, Martine Hoogman

**Affiliations:** 1 Department of Psychiatry, Radboud University Medical Center, Nijmegen, The Netherlands; 2 Department of Human Genetics (855), Radboud University Medical Center, PO Box 9101, 6500 HB Nijmegen, The Netherlands; 3 Donders Institute for Brain, Cognition and Behaviour, Radboud University, Nijmegen, The Netherlands; 4 Clinical Neuropsychology Section, Department of Clinical Psychology, VU University Amsterdam, Amsterdam, The Netherlands; 5 Department of Psychiatry, University of Groningen, University Medical Center Groningen, Groningen, The Netherlands; 6 Department of Cognitive Neuroscience, Radboud University Medical Center, Nijmegen, The Netherlands; 7 Karakter Child and Adolescent Psychiatric University Centre, Nijmegen, The Netherlands

**Keywords:** ADHD, *DAT1* gene, Striatum, Volumetry

## Abstract

The dopamine transporter gene, DAT1 (*SLC6A3*), has been studied extensively as a candidate gene for attention-deficit/hyperactivity disorder (ADHD). Different alleles of variable number of tandem repeats (VNTRs) in this gene have been associated with childhood ADHD (10/10 genotype and haplotype 10-6) and adult ADHD (haplotype 9-6). This suggests a differential association depending on age, and a role of *DAT1* in modulating the ADHD phenotype over the lifespan. The *DAT1* gene may mediate susceptibility to ADHD through effects on striatal volumes, where it is most highly expressed. In an attempt to clarify its mode of action, we examined the effect of three *DAT1* alleles (10/10 genotype, and the haplotypes 10-6 and 9-6) on bilateral striatal volumes (nucleus accumbens, caudate nucleus, and putamen) derived from structural magnetic resonance imaging scans using automated tissue segmentation. Analyses were performed separately in three cohorts with cross-sectional MRI data, a childhood/adolescent sample (NeuroIMAGE, 301 patients with ADHD and 186 healthy participants) and two adult samples (IMpACT, 118 patients with ADHD and 111 healthy participants; BIG, 1718 healthy participants). Regression analyses revealed that in the IMpACT cohort, and not in the other cohorts, carriers of the *DAT1* adult ADHD risk haplotype 9-6 had 5.9 % larger striatum volume relative to participants not carrying this haplotype. This effect varied by diagnostic status, with the risk haplotype affecting striatal volumes only in patients with ADHD. An explorative analysis in the cohorts combined (*N* = 2434) showed a significant gene-by-diagnosis-by-age interaction suggesting that carriership of the 9-6 haplotype predisposes to a slower age-related decay of striatal volume specific to the patient group. This study emphasizes the need of a lifespan approach in genetic studies of ADHD.

**Electronic supplementary material:**

The online version of this article (doi:10.1007/s00702-016-1521-x) contains supplementary material, which is available to authorized users.

## Introduction

Attention-deficit/hyperactivity disorder (ADHD) is a common childhood-onset psychiatric disorder that features symptoms of age-inappropriate inattention and/or impulsivity and hyperactivity. ADHD affects 5–6 % of children (Polanczyk et al. 2007) and frequently persists into adulthood (Faraone et al. 2006) causing a prevalence of ADHD of between 2.5 and 4.9 % in the adult population (Simon et al. 2009). The heritability of ADHD is around 0.8 in both children (Faraone et al. 2005) and adults (Larsson et al. 2013). ADHD’s complex genetic etiology likely involves multiple genes of small to moderate effect (Akutagava-Martins et al. 2013).

The dopamine neurotransmission system has been an important focus of genetic research in ADHD, since it is the main site of action of stimulant drugs, the primary pharmacological treatment for the disorder (Cortese 2012; Faraone et al. 2014a). One of the most appealing and extensively studied candidate genes for ADHD is the dopamine transporter (*DAT1*) gene (official name *SLC6A3*) (Faraone et al. 2005; Franke et al. 2012). The dopamine transporter is a key determinant of synaptic dopamine levels by regulating the reuptake of dopamine from the extracellular space, thereby terminating its synaptic action (Madras et al. 2005). The association between *DAT1* and ADHD was suggested in linkage and association studies and is confirmed in meta-analyses (Franke et al. 2010; Gizer et al. 2009; Li et al. 2006) showing small but significant effects on the susceptibility to ADHD. Meta-analyses of genetic association studies have indicated that the 10-repeat allele of the 3′ untranslated region (UTR) variable number of tandem repeat (VNTR) is overrepresented in children with ADHD (Gizer et al. 2009). More recent studies suggested that the 10-repeat allele might increase ADHD risk in children particularly in the context of a haplotype with the 6-repeat allele of another VNTR in intron 8 of the gene (Asherson et al. 2007; Brookes et al. 2008). A recent study also found an association between this 10-6 haplotype and ADHD symptom measures in nonclinical adults (Tong et al. 2015), but association studies in clinical samples of adults with ADHD could not confirm this relationship (Brüggemann et al. 2007) and reported an association of the 9-6 haplotype with adult ADHD (Franke et al. 2008, 2010). Together, these findings suggest a role for *DAT1* in modulating the ADHD phenotype across the lifespan, with different associations depending on age and diagnostic status.

The specific mechanisms by which *DAT1* genetic variants affect the risk for ADHD are not well understood. Two imaging genetics studies showed that genetic variation of the *DAT1* gene is associated with altered striatal volume, which may contribute to ADHD susceptibility; the caudate nucleus, a sub-region of the striatum, was found to be smaller in children homozygous for the 10-repeat allele (10/10) than in carriers of the 9-repeat allele (Durston et al. 2005; Shook et al. 2011). Although both studies did not found an interaction between presence/absence of ADHD and genotype, Durston et al. (2005) reported that the effect of *DAT1* genotype on caudate volume was only significant in the subgroup of patients with ADHD. Studies investigating the effect of the *DAT1* gene on prefrontal gray matter volume, cortical thickness, or white matter integrity found no association between 10-repeat allele carriers (10/10) and 9-repeat allele carriers (Durston et al. 2005; Hong et al. 2015; Shaw et al. 2007), suggesting that this gene primarily affects regions, where it is highly expressed (i.e., the striatum) (Ciliax et al. 1999; Durston et al. 2009).

The effect of the *DAT1* gene on striatal volumes may help explain smaller volumes of caudate nucleus and putamen typically found in children with ADHD (Ellison-Wright et al. 2008; Frodl and Skokauskas 2012; Nakao et al. 2011; Valera et al. 2007). It has been shown that volumetric differences in caudate nucleus and the putamen gradually disappear with age (Castellanos et al. 2002; Frodl and Skokauskas 2012; Greven et al. 2015; Maier et al. 2015; Nakao et al. 2011). The largest study to date by the ENIGMA ADHD Working Group containing 1713 participants with ADHD and 1529 controls show (among others) reduced accumbens, caudate nucleus, and putamen volume in ADHD. Case–control differences were most pronounced in childhood confirming a model of delayed brain growth and maturation (Hoogman et al., submitted). Nonetheless, there is evidence from studies of adults with persistent ADHD that differences in caudate nucleus volume (Almeida Montes et al. 2010; Onnink et al. 2014; Proal et al. 2011; Seidman et al. 2011; Shaw et al. 2014) and putamen volume (Seidman et al. 2011; Shaw et al. 2014) persist into adulthood.

To summarize, existing literature points to different alleles of the *DAT1* increasing susceptibility to categorically defined ADHD from childhood to adulthood, with a possible role of striatal volume in the pathway from gene to disease. The evidence for an influence of *DAT1* on striatal volume is based on relatively small-sampled studies [*N* = 59 in Shook et al. (2011) and *N* = 72 in Durston et al. (2005)]. Moreover, these studies examined only one variant of the *DAT1* gene (10/10 homozygotes versus 9-repeat carriers), not taking into account the potentially stronger effects of the two-VNTR haplotypes. Importantly, they were conducted in children only and could not test possible different effects of gene variation on striatal volume across the lifespan.

In the current study, we therefore set out to investigate the effects of the three different *DAT1* risk variants on striatal brain volume (nucleus accumbens, caudate nucleus, putamen) and the potential interaction with diagnostic status and age. We defined the *DAT1* 10/10 genotype, the 10-6 haplotype, and the 9-6 haplotype as risk alleles, based on associations with ADHD in children (10/10 genotype and 10-6 haplotype) and in adults (9-6 haplotype), respectively. Participants were derived from three cohorts with cross-sectional MRI data, a childhood/adolescent sample (NeuroIMAGE, 301 patients with ADHD and 186 healthy controls) and two adult samples (IMpACT, 118 patients with ADHD and 111 healthy controls; BIG, 1718 healthy participants).

## Methods

### Participants

Participants of this study were derived from three distinct cohorts. Ethical approval for all three was obtained, and all participants provided written informed consent.

A total of 487 subjects (301 unrelated patients with ADHD and 186 control participants) were derived from the NeuroIMAGE cohort of families with ADHD and control families (http://www.neuroimage.nl) (von Rhein et al. 2015). Only one individual per family was included thus (un)affected siblings were not included in this study. Participants were recruited at VU University Amsterdam, Amsterdam, and Radboud University Medical Center, Nijmegen. Inclusion criteria were an age between 8 and 30 years; European Caucasian descent; intelligence quotient (IQ) greater than or equal to 70; and no diagnosis of autism, epilepsy, general learning difficulties, brain disorders, and known genetic disorders. All participants were evaluated with a semi-structured diagnostic interview assessing ADHD, oppositional defiance disorder (ODD), and conduct disorder (CD). For further details on diagnostic assessment, see von Rhein et al. (2015).

A total of 229 subjects (118 adult patients with ADHD and 111 control participants) were included from the Dutch cohort of the International Multicentre persistent ADHD CollaboraTion, IMpACT (http://www.impactadhdgenomics.com; (Franke et al. 2010; Onnink et al. 2014). Participants were recruited at Radboud University Medical Center, Nijmegen. All participants were evaluated with semi-structured diagnostic interviews for assessing ADHD and axis I and axis II disorders. For details on diagnostic assessment, see Onnink et al. (2014). Inclusion criteria were an age between 18 and 65 years; European Caucasian descent; IQ greater than or equal to 70; no diagnosis of psychosis, alcohol or substance use disorder in the last 6 months, current major depression, neurological and sensorimotor disorders. An exclusion criterion for the control participants was a current neurological or psychiatric disorder.

A total of 1718 control participants were included from the Cognomics Initiative Resource, the Brain Imaging Genetics (BIG) study (http://www.cognomics.nl). This ongoing study started in 2007 and is a collection of healthy volunteers, many with a high education level, who participated in studies at the Donders Centre for Cognitive Neuroimaging (DCCN) of the Radboud University in Nijmegen (Guadalupe et al. 2014). The self-reported healthy individuals underwent anatomical (T1-weighted) magnetic resonance imaging (MRI) scans, usually as part of their involvement in diverse smaller-scale studies at the DCCN.

### Genotyping

In all three cohorts, DNA was isolated from EDTA blood samples or saliva samples using standard procedures. Genotyping of the 40 base pair VNTR in the 3′UTR and the VNTR in intron 8 of *DAT1/SLC6A3* was carried out at the department of Human Genetics of the Radboud University Medical Center, Nijmegen as is described earlier (Franke et al. 2010). Haplotypes were calculated using the Haplostats package (Rversion 2.12.0) (Schaid et al. 2002).

### Image acquisition and segmentation

MRI data in NeuroIMAGE were acquired at two locations (VU University Amsterdam, Amsterdam, and Radboud University Medical Center, Nijmegen) using two similar 1.5 Tesla (T) scanners (Sonata and Avanto; Siemens Medical Systems, Erlangen, Germany) with closely matched scan protocols (von Rhein et al. 2015). MRI data in IMpACT were acquired with a 1.5T scanner (Avanto; Siemens Medical Systems, Erlangen, Germany). For NeuroIMAGE, GRAPPA2 (generalized autocalibrating partial parallel acquisition) and for IMpACT magnetization prepared rapid gradient echo sequence (MPRAGE) sequences were used. For NeuroIMAGE and IMpACT, all scans covered the entire brain and had a voxel size of 1 × 1 × 1 mm (176 sagittal slices; repetition time = 2730 ms; echo time = 2.95 ms; inversion time = 1000 ms; flip angle = 7°; field of view = 256 mm). MRI data in BIG were acquired with either a 1.5T (Sonata and Avanto; Siemens Medical Systems, Erlangen, Germany) (*N* = 923) or with a 3T Siemens scanner (Trio and TimTrio; Siemens Medical Systems, Erlangen, Germany) (*N* = 796). Given that images were acquired during several smaller scale studies, the parameters used were slight variations of a standard T1-weighted sequence (MPRAGE; voxel size of 1 × 1 × 1 mm). The most common variations in the TR/TI/TE/saggital-slices parameters were the following: 2300/1100/3.03/192, 2730/1000/2.95/176, 2250/850/2.95/176, 2250/850/3.93/176, 2250/850/3.68/176, 2300/1100/3.03/192, 2300/1100/2.92/192, 2300/1100/2.96/192, 2300/1100/2.99/192, 1940/1100/3.93/176 and 1960/1100/4.58/176. Such slight variations in these imaging parameters have been shown not to affect the reliability of morphometric results (Jovicich et al. 2009).

### Whole-brain volume

Normalization, bias correction, and segmentation into gray matter, white matter, and cerebrospinal fluid volumes were performed using the unified procedure of the VBM 8.1 toolbox (http://dbm.neuro.uni-jena.de/vbm/) in SPM (default settings). Total gray and white matter volumes were calculated by summation of their tissue probability maps. Total brain volume was the sum of total gray and white matter volumes.

### Striatal volumes

Automated FIRST (FMRIB’s Integrated Registration and Segmentation Tool) subcortical segmentation was applied to estimate left and right hemisphere volumes of the nucleus accumbens, caudate nucleus, and putamen. The ENIGMA protocol (http://enigma.ini.usc.edu/protocols/imaging-protocols/) for the FIRST module (version 1.2) of FSL (version 4.1.5) was followed. FIRST is part of FMRIB’s Software Library and performs registration and shape modeling of the just-mentioned regions in Montreal Neurological Institute 152 standard space (Patenaude et al. 2011). Total striatal volume was the sum of left and right volumes of the nucleus accumbens, caudate nucleus, and putamen.

### Statistical analyses

Brain volumetric measures were normally distributed, and outliers defined as more than three standard deviations greater than or less than the mean were removed. Overall, there were few outliers (1–5 individuals per volume). For each cohort independently, the effect of three variants of the *DAT1* gene on striatal volumes were examined by comparing: (1) carriers of the 10/10 genotype with all non-carriers, (2) carriers of at least one copy of the 10-6 haplotype with all non-carriers, and (3) carriers of at least one copy of the 9-6 haplotype with all non-carriers. Associations between the three risk variants of the *DAT1* gene and striatal volumes were examined using regression analyses in SPSS (IBM SPSS v.20). Regression analyses included variant of the *DAT1* gene, diagnostic status, and the interaction between risk variant and diagnostic status (*DAT1* variant × diagnostic status) as predictors and total striatal volume as dependent measure. Included covariates were age, gender, and total brain volume (sum of white and gray matter); for the NeuroIMAGE and BIG cohorts, additional covariates were scanner location and type (for NeuroIMAGE: Amsterdam or Nijmegen; for BIG: 1.5T or 3.0T); for the BIG cohort with healthy participants, diagnostic status was dropped from the model. Centering of variables was used (Bradley and Srivastava 1979). First, we tested the interaction between *DAT1* variant and diagnostic status. Whenever this interaction term was significant (*p* < .05), we analyzed the results separately by diagnostic status. If not significant, this interaction was dropped from the model. For significant main effects of the three risk variants, we performed post hoc sensitivity analyses. Correcting with covariates in a regression analysis is only appropriate if covariate means or distributions are equal between groups (Miller and Chapman 2001). Therefore, sensitivity analyses in a matched subsample were performed for the instances in which covariates differed between groups. Automatic case–control matching was performed with the FUZZY extension for SPSS (http://www.spss.com/devcentral). Sensitivity analyses were performed to investigate the effect of the risk variant on each subregion of the striatum (left and right volumes of nucleus accumbens, caudate nucleus, and putamen). Additionally, we investigated the possible effect of medication on the results by including lifetime medication use (yes or no) to the model. To explore potential interactions between *DAT1* variant, diagnostic status, and age on striatal volume (*DAT1* variant × diagnostic status × age), we combined the samples from the three cohorts into one sample in order to maximize the age range. Then, striatal volume was adjusted for the same covariates as mentioned above, except age, using a linear regression analysis from which standardized residuals were computed and were used in the analyses (Walhovd et al. 2005). To visualize potential age effects, the residuals were also plotted.

### Correction for multiple testing

To correct for multiple testing, Bonferroni correction was applied by dividing the significance level by the number of independent tests. In three cohorts (NeuroIMAGE, IMpACT, BIG), we examined the effects of three alleles/genotypes (10/10, 10-6 haplotype, 9-6 haplotype) on striatal volume. We performed a total of nine tests and set the multiple-testing adjusted *p* value at 0.05/9 = 0.0055. Post-hoc sensitivity analyses of findings surviving multiple-testing correction used the nominal significance level (*p* < .05).

## Results

### Demographics

Demographics for ADHD patients and control participants are displayed for the NeuroIMAGE, IMpACT, and BIG cohorts separately in Table 1. From the NeuroIMAGE cohort, the 301 patients with ADHD and 186 control participants were evenly distributed across groups based on VNTR genotypes (10/10) and *DAT1* haplotypes (10-6 haplotype or 9-6 haplotype). In this cohort, patients were significantly older compared with the control participants [*t*(1, 485) = 2.21, *p* = .03], and gender distribution was significantly different, with males predominating in the ADHD group and females in the control group (*χ*^2^ = 16.19, *p* < .001). From the IMpACT cohort, 118 patients with ADHD and 111 control participants were included, for which no differences in the distribution of *DAT1* 10/10 genotype and *DAT1* 10-6 haplotype were observed. The 9-6 haplotype showed a higher prevalence in patients compared with controls (*χ*^2^ = 5.21, *p* = .023; see Table 1), as was reported previously in this cohort (Hoogman et al. 2012). From the BIG cohort, 1718 healthy participants were included. Genotype distributions did not deviate from Hardy–Weinberg equilibrium, and frequencies were as expected in Caucasian samples (Franke et al. 2010).

**Table 1:** Participant characteristics for the three cohorts included in this study

Characteristics	NeuroIMAGE (*N* = 487)		Test of significance	IMpACT (*N* = 229)		Test of significance	BIG (*N* = 1718)
	ADHD (*N* = 301)	Controls (*N* = 186)		ADHD (*N* = 118)	Controls (*N* = 111)		Controls (*N* = 1718)
10/10 carriers, *N* (%)	183 (61)	105 (56)	*χ* ^2^ = 0.90, *p* = .34	61 (52)	69 (62)	*χ* ^2^ = 2.55, *p* = .11	978 (57)
10-6 carriers, *N* (%)	282 (94)	174 (94)	*χ* ^2^ = 0.01, *p* = .95	107 (91)	102(92)	*χ* ^2^ = 0.11, *p* = .75	1573 (92)
9-6 carriers, *N* (%)	49 (16)	24 (13)	*χ* ^2^ = 1.03, *p* = .31	26 (22)	12 (11)	*χ* ^2^ = 5.21, *p* = .02	249 (14)
Male, *N* (%)	207 (69)	94 (51)	*χ* ^*2*^ = 16.19, *p* < .001	46 (39)	46 (41)	*χ* ^2^ = 0.14, *p* = .71	749 (44)
Age in years, mean (SD)	17.21 (3.27)	16.55 (3.06)	*t*(1, 485) = −2.21, *p* = .03	35.94 (10.93)	37.03 (11.28)	*t*(1, 227) = 0.72, *p* = .47	26.06 (10.63)
IQ, mean (SD)	97.02 (15.24)	106.39 (13.38)	*t*(1, 485) = 6.89, *p* < .001	107.81 (14.50)	110.03 (15.41)	*t*(1, 227) = 1.12, *p* = .26	nd
Inattentive scale, mean (SD)^a^	65.89 (11.09)	46.28 (5.70)	*t*(1, 485) = −22.27, *p* < .001	6.46 (2.04)	0.66 (1.12)	*t*(1, 227) = −26.81, *p* < .001	1.20 (1.66)
Hyperactive/impulsive scale, mean (SD)^a^	69.63 (14.45)	46.28 (5.01)	*t*(1, 485) = −21.19, *p* < .001	5.48 (2.24)	0.90 (1.38)	*t*(1, 227) = −18.49, *p* < .001	1.62 (1.65)
Total brain volume in ml, mean (SD)^b^	1257.73 (125.41)	1265.41 (123.03)	*t*(1, 485) = 0.61, *p* = .51	1255.06 (106.58)	1240.83 (124.09)	*t*(1, 227) = −0.93, *p* = .35	123.90 (120.10)

*nd* not determined

^a^For NeuroIMAGE cohort: measured with the Conners’ parent rating scale—revised (Conners et al. 1998). Values refer to *t* scores on the *DSM* total, inattentive behavior, and hyperactive-impulsive behavior scales (scales N, L, and M). For IMpACT and BIG cohorts: measured with the ADHD-DSM-IV self rating scale (Kooij et al. 2005)

^b^Total brain volume is defined as the sum of total gray and white matter

Demographics for 10/10, 10-6, and 9-6 carrier and respective non-carrier groups are displayed for the NeuroIMAGE, IMpACT, and BIG cohorts separately in supplementary Tables 1, 2, and 3. In the IMpACT cohort, gender distribution was significantly different between *DAT1* 10/10 carriers and non-carriers (*χ*^2^ = 4.47, *p* = .03; supplementary Table 1), with males predominating in the *DAT1* 10/10 group and females in the non-*DAT1* 10/10 group. Gender distribution was also significantly different between *DAT1* 9-6 carriers and non-carriers (*χ*^2^ = 5.16, *p* = .02; supplementary Table 3), with males predominating in the *DAT1* 9-6 group and females in the non-carriers.

### Main and interaction effects of *DAT1* variants on total striatum volume

For each cohort, mean total striatum volumes corrected for covariates are shown in Table 2. In the IMpACT cohort, subjects carrying at least one copy of the 9-6 risk haplotype showed a 5.9 % larger striatum volume (1.09 ml larger) than subjects carrying none (*β* = 1.09; 95 % CI 0.63–1.56; *p* = .00001) (Tables 2, 3). No effects of the *DAT1* variant (combinations) were observed in the NeuroIMAGE or BIG cohorts.

**Table 2:** Total striatum volume for risk and non-risk carriers of specific *DAT1* variants

	Total striatum volume (ml)						
	NeuroIMAGE (*N* = 487)			IMpACT (*N* = 229)			BIG (*N* = 1718)
	Overall^a^	Controls^b^	ADHD^b^	Overall^a^	Controls^b^	ADHD^b^	Overall^b^
	Mean (SE), *N*	Mean (SE), *N*	Mean (SE), *N*	Mean (SE), *N*	Mean (SE), *N*	Mean (SE), *N*	Mean (SE), *N*
10/10 carriers	20.23 (0.09), 288	20.11 (0.13), 105	20.33 (0.10), 183	18.84 (0.12), 130	19.10 (0.16), 69	18.55 (0.17), 61	19.42 (0.04), 977
Non-carriers	20.01 (0.10), 199	20.15 (0.15), 81	19.96 (0.13), 118	19.01 (0.14), 99	18.74 (0.21), 42	19.19 (0.18), 57	19.45 (0.05), 741
10-6 carriers	20.11 (0.07), 456	20.11 (0.10), 174	20.16 (0.08), 282	18.87 (0.09), 209	19.11 (0.45), 102	18.81 (0.13), 107	19.44 (0.03), 1572
Non-carriers	20.46 (0.25), 31	20.32 (0.39), 12	20.60 (0.32), 19	19.28 (0.31), 20	18.95 (0.13), 9	19.34 (0.43), 11	19.42 (0.10), 146
9-6 carriers	19.95 (0.17), 73	20.02 (0.27), 24	20.01 (0.21), 49	**19.83 (0.22), 38**	19.47 (0.38), 12	**19.93 (0.25), 26**	19.49 (0.08), 249
Non-carriers	20.17 (0.07), 414	20.14 (0.11), 162	20.22 (0.09), 301	**18.73 (0.09), 191**	18.90 (0.13), 99	**18.56 (0.13), 92**	19.43 (0.03), 1469

Volumes are also shown for controls and ADHD patients, separately

Total striatum volume is the sum of total left and right nucleus accumbens, caudate nucleus, and putamen volumes

Boldface indicates results surviving multiple-testing correction

^a^Means are based on estimated marginal means corrected for diagnostic status, age, gender, total brain volume; for the NeuroIMAGE cohort, covariates also included scanner type

^b^Means are based on estimated marginal means corrected for age, gender, total brain volume; for the NeuroIMAGE and BIG cohorts, covariates also included scanner type/location

**Table 3:** Regression of binary genotypes on total striatal volume

	NeuroIMAGE (*N* = 487)	IMpACT (*N* = 229)	BIG (*N* = 1718)
	*β* (95 % CI), *p* value^a^	*β* (95 % CI), *p* value^a^	*β* (95 % CI), *p* value^a^
*DAT1* 10/10	0.22 (−0.04; 0.48), .09	−0.16 (−0.53; 0.20), .38	−0.03 (−0.15; 0.09), .57
Diagnostic status	0.22 (−0.05; 0.49), .12	−0.29 (−0.62; 0.07), .11	
Diagnostic status × *DAT1* 10/10	ns	−1.03 (−1.74; −0.32), .005	
*DAT1* 10-6	−0.35 (−0.86; 0.16), .18	−0.41 (−1.04; 0.22), .21	0.02 (−0.19; 0.24), .84
Diagnosis	0.24 (−0.34; 0.51), .09	−0.28 (−0.63; 0.08), .13	
Diagnostic status × *DAT1* 10-6	ns	ns	
*DAT1* 9-6	−0.22 (−0.57; 0.13), .21	1.09 (0.63; 1.56), **.00001** ^b^	0.06 (−0.11; 0.23), .47
Diagnosis	0.24 (−0.33; 0.51), .09	−0.40 (−0.74; −0.05), .024	
Diagnostic status × *DAT1* 9-6	ns	1.14 (0.17; 2.11), .021	

For the NeuroIMAGE and IMpACT cohorts, interactions with genotype and diagnostic status (genotype × diagnostic status) were tested and removed when not nominal significant (*p* < .05)

Results from the final regression model examining associations between binary genotype (risk carriers vs non-risk carriers) and brain volumes. Boldface indicates results surviving multiple-testing correction

*ns* not significant

^a^For main effects, *β* (unstandardized regression coefficient) is equal to the difference in mean brain volumes (in ml) between the genotype groups adjusted for covariates in the model. Included covariates were diagnostic status, age, gender, total brain volume; for the NeuroIMAGE and BIG cohorts, covariates also included scanner type; for the BIG cohort, diagnosis was dropped from the model

^b^ *β* = 1.09 denotes that 9-6 carriers had a 1.09 ml larger striatum volume than non 9-6 carriers

In the IMpACT cohort, an interaction between the *DAT1* 9-6 haplotype and diagnostic status on striatal volume was significant (*p* = .02). Testing patients with ADHD and controls separately revealed that patients carrying at least one copy of the *DAT1* 9-6 haplotype had larger striatum volume (7.4 %; 1.37 ml; *β* = 1.37; 95 % CI 0.80–1.94; *p* = .00001), while this effect was not significant in the control group (3.0 %; 0.57 ml; *β* = 0.57; 95 % CI −0.25 to 1.39; *p* = .17) (Table 3 and supplementary Table 5). Another significant interaction also observed in the IMpACT cohort was between diagnostic status and *DAT1* 10/10 genotype (*p* = .005). Post-hoc analyses revealed that patients homozygous for the 10R allele (10/10 carriers) had smaller striatum volume than 9R carriers (−3.5 %; 0.64 ml; *β* = −0.64; 95 % CI −1.14 to −0.14; *p* = .013), while this effect was not present in the control group (1.4 %; 0.36 ml; *β* = 0.36; 95 % CI −0.17 to 0.89; *p* = .18) (Table 3 and supplementary Table 4).

### Sensitivity analyses

In the NeuroIMAGE cohort, gender distribution and age were significantly different between patients and controls (Table 1). We therefore examined the effect of the three variants of the *DAT1* gene on striatal volume in a subample that was matched for gender and age (supplementary Table 6). The results in this matched subsample (supplementary Table 7) supported the results of the unmatched sample (Table 3). In the IMpACT cohort, gender distribution was significantly different between *DAT1* 10/10 carriers and non-carriers (supplementary Table 1) and between *DAT1* 9-6 carriers and non-carriers (supplementary Table 3). However, analysis of the effects of these two *DAT1* variants on striatal volume in a gender-matched subsample (supplementary Table 8) confirmed the results observed in the full sample (supplementary Table 9 and Table 3). The effect of the *DAT1* 9-6 haplotype on striatal volume found in the IMpACT cohort was the strongest effect observed, surviving multiple-testing correction, and was investigated further. Sensitivity analyses in the IMpACT cohort were performed to examine the effect of the *DAT1* 9-6 haplotype on the six subregions of the striatum independently (left and right volumes of nucleus accumbens, caudate nucleus, and putamen). Compared to subjects carrying no copies of the 9-6 risk haplotype, subjects carrying at least one copy of the risk haplotype had larger right putamen volume (6.2 %; 0.33 ml; *β* = 0.33; 95 % CI 0.17–0.48; *p* = .00005), larger left putamen (6.1 %; 0.32 ml; *β* = .32; 95 % CI 0.14–0.49; *p* = .0004), larger right caudate nucleus (5.9 %; 0.22 ml; *β* = 0.22; 95 % CI 0.09–0.35; *p* = .001), larger left caudate nucleus (5.5 %; 0.20 ml; *β* = 0.20; 95 % CI 0.07–0.33; *p* = .002), and larger right nucleus accumbens (5.8 %; 0.03 ml; *β* = 0.03; 95 % CI 0.01–0.06; *p* = .04). Findings were not significant for left nucleus accumbens (*p* > .05) (supplementary Table 10). Testing the effect of the *DAT1* 9-6 haplotype on the six subregions of the striatum for patients with ADHD and controls separately revealed similar results as above in the patients, while effects were non-significant in controls (all *p* values >.05) (supplementary Table 11). Furthermore, rerunning analyses including medication use (yes or no) in the model yielded highly similar results (supplementary Table 12).

### Age effects of the *DAT1* 9-6 haplotype

**Fig. 1:**
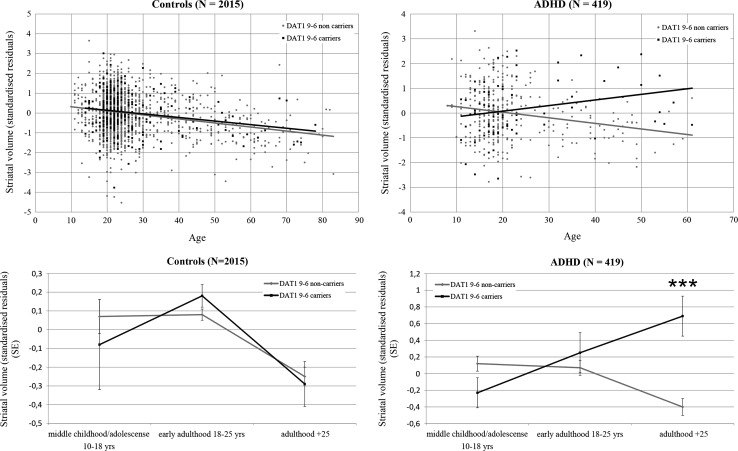
Age-related changes in the striatal volume. **a** Regression plots visualizing the 3-way interaction (*DAT1* genotype × diagnostic status × age) by plotting the relationships between age and total striatal volume for *DAT1* 9-6 haplotype carriers and non-carriers separately for controls and ADHD patients. **b** Same data as in** a** although now visualized using separate age groups. The figure suggests that carriership of the 9-6 haplotype predisposes to a slower age-related decay of striatal volume in patients with ADHD

To explore potential interactions between the *DAT1* 9-6 haplotype, diagnostic status, and age on total striatum volume, we combined the samples from the three cohorts into one sample in order to maximize the age range. Total striatal volume was regressed on covariates of no interest and the standardized residuals were used for analysis. In this mega-analysis design, the 3-way interaction between the 9-6 haplotype, diagnostic status, and age on striatal volume was significant (*p* = .0001). Testing patients with ADHD and controls separately revealed that the interaction between *DAT1* 9-6 haplotype and age was significant in the patient group (*p* = .00004) but not in the control group (*p* = .94) (Fig. 1).

## Discussion

In the current study, the effect of the dopamine transporter gene *DAT1*/*SLC6A3* on striatal brain volume was investigated in children and adults with ADHD and healthy participants in three different cross-sectional cohorts. In the adult case–control cohort IMpACT, carriers of the 9-6 haplotype, the risk allele for adult ADHD, had larger striatal volume than participants not carrying this haplotype. This effect varied by diagnostic group, with the risk haplotype affecting striatal volumes only in patients with ADHD and not in the healthy participants from this cohort. Consistent with this, the effect was not found in the BIG cohort of adult healthy participants. It was also not observed in the case–control children/adolescents cohort from NeuroIMAGE. Through an interaction analysis within the IMpACT cohort, also the 10/10 genotype was shown to affect striatal volume in patients only when compared to carriers of 9R allele(s), which was a smaller effect than for the 9-6 haplotype (and probably was just the other side of the same coin).

The finding in the IMpACT cohort showing smaller striatal volume in adult ADHD patients homozygous for the 10R allele (10/10 carriers) compared to 9R carriers is consistent with previous studies performed in children (Durston et al. 2005; Shook et al. 2011). However, as 84 % of the 9R carriers consisted of 9-6 haplotype carriers, this effect might be driven by the subgroup of 9-6 haplotype carriers. Indeed, the regression coefficient of −0.64 (*p* = .013, *N* = 118) (supplementary Table 4) dropped to −0.074 (*p* = .78, *N* = 92) when the 9-6 haplotype carriers (*N* = 26) were excluded from the analysis (data not shown). The diagnosis-specificity of *DAT1* only affecting striatal volume in the subgroup of patients with ADHD was also suggested in the previous study by Durston et al. (2005). Larger striatal volume in adult carriers of the *DAT1* risk haplotype 9-6 for adult ADHD may represent compensatory mechanisms for the increased expression/activity of the dopamine transporter, which has been found in 9-repeat allele carriers (Faraone et al. 2014b). The increased levels of DAT in these individuals might lead to more efficient clearing of extracellular dopamine, yielding lower extracellular levels and reduced dopamine signaling (Faraone et al. 2014b). Importantly, a study by Spencer and coworkers showed that an ADHD diagnosis made an additional, independent contribution to DAT binding (Spencer et al. 2013). The diagnosis-specificity of our findings may thus reflect an interaction between genetic and environmental risk factors, where cumulative effects allow for a bigger impact of *DAT1* genotype on striatal volume in the patients. We emphasize, nonetheless, that replication of our findings is needed before firm conclusions can be drawn.

Our explorative 3-way interaction analysis in the cohorts combined (*N* = 2434) investigating the effect *DAT1* 9-6 haplotype, diagnostic status, and age suggests that carriership of the 9-6 haplotype predisposes to a slower age-related decay of striatal volume, which is specific for ADHD patients (Fig. 1). Importantly, age effects have shown a differential decay of *DAT1* expression for different genotypes (Shumay et al. 2011), which may be consistent with the compensation hypothesis mentioned above. Shumay et al. demonstrated that 9-repeat homozygotes showed the steepest decline of DAT availability with increasing age. Great care is needed in interpreting the age effects we observed, as this is a cross-sectional study. Interestingly, a recent study suggests that individuals can meet symptom criteria for ADHD as adults without having a history of childhood ADHD (Moffitt et al. 2015). Although this study by Moffitt et al. is in need of replication, our results may suggest that carriership of the *DAT1* 9-6 haplotype might be a mechanism contributing to the emergence of new cases of ADHD during adulthood. However, to replicate our age-dependent effect and to explore this more fully, analysis of longitudinal MRI data is required.

The functional implications of larger striatal volume for the pathophysiology of adult ADHD remain to be investigated. As smaller caudate volume in male patients with ADHD has been associated with an increased number of hyperactivity/impulsivity symptoms (Onnink et al. 2014), larger striatum volume in a subgroup of ADHD patients may be linked to neurobiological processes that go along with the reported age-dependent decline in hyperactivity/impulsivity symptoms in people with ADHD (Biederman et al. 2000). Increased volume may also reflect compensatory ‘hypertrophy’ because of reduced dopamine neurotransmission (see above).

Our findings should be viewed in the light of certain strengths and limitations. A clear strength was the investigation of haplotypes of *DAT1* in addition to the 3′UTR VNTR genotype variants in a large sample including patients with ADHD and healthy individuals at different ages. This case–control design maximized the variance in the phenotype and may have magnified gene effects. A strong limitation was the cross-sectional MRI study design, especially since the participants of this study were partly derived from different cohorts. Another limitation was the restricted availability of data at early childhood age and late adult age, which reflects insufficient focus of imaging research in our field on such age groups. The developmental trajectories our data propose need to be confirmed in additional studies, optimally from longitudinal studies including data across a wide age range collected using the same study protocol.

In summary, our cross-sectional findings showed that adult patients with ADHD carrying the *DAT1* 9-6 risk haplotype for adult ADHD had increased striatal volume. Furthermore, based on our exploratory analysis on age effects, we hypothesize that ADHD patients carrying the 9-6 haplotype follow a different trajectory of brain development over the lifespan than those ADHD patients not carrying this haplotype. These findings are in need of replication, preferably using longitudinal designs. Clarifying the nature of the involvement of *DAT1* variants in brain development would provide a key step towards understanding part of ADHD’s pathophysiology. The present results demonstrate the importance of taking into account interindividual variability, as indexed by *DAT1* haplotype, presence of an ADHD diagnosis, and age, when assessing striatal volume effects in ADHD.

## Electronic supplementary material

Below is the link to the electronic supplementary material.

Supplementary material 1 (DOCX 22 kb)

Supplementary material 2 (DOCX 22 kb)

Supplementary material 3 (DOCX 20 kb)

Supplementary material 4 (DOCX 17 kb)

Supplementary material 5 (DOCX 17 kb)

Supplementary material 6 (DOCX 19 kb)

Supplementary material 7 (DOCX 18 kb)

Supplementary material 8 (DOCX 20 kb)

Supplementary material 9 (DOCX 18 kb)

Supplementary material 10 (DOCX 17 kb)

Supplementary material 11 (DOCX 18 kb)

Supplementary material 12 (DOCX 16 kb)
