# Supplementary material for: Enlarged striatal volume in adults with ADHD carrying the 9-6 haplotype of the dopamine transporter gene DAT1
Source: J Neural Transm (Vienna). 2016 Mar 2;123:905–15. doi: 10.1007/s00702-016-1521-x (PMC4969340; doi:10.1007/s00702-016-1521-x)
Supplement: Supplementary file 1 — Supplementary material 1 (DOCX 22 kb) [file 702_2016_1521_MOESM1_ESM.docx]

Supplementary Table 1. Participant characteristics for the *DAT1* 10/10 carriers and non-carriers for the three cohorts included in this study.

|  | NeuroIMAGE (N = 487) | | | IMpACT (N = 229) | | | BIG (N = 1718) | | |
| --- | --- | --- | --- | --- | --- | --- | --- | --- | --- |
| Characteristics | *DAT1* 10/10 carriers  (N = 288) | *DAT1* 10/10 non-carriers  (N = 199) | Test of significance | *DAT1* 10/10 carriers  (N = 130) | *DAT1* 10/10 non-carriers  (N = 99) | Test of significance | *DAT1 10/10* carriers  (N = 977) | *DAT1 10/10* non-carriers (N = 741) | Test of significance |
| Male, N (%) | 167 (58) | 134 (67) | χ^2^ = 4.36, *p* = .05 | 60 (46) | 32 (32) | χ^2^ = 4.47, *p* = .03 | 416 (43) | 333 (45) | χ^2^ = 0.95, *p* = .33 |
| Age in years, mean (SD) | 16.99 (3.29) | 16.91 (3.08) | t(1, 485) =  0.26, *p* = .80 | 35.77 (10.95) | 37.38 (12.29) | t(1, 227) = 1.07, *p* = .29 | 26.33 (11.19) | 25.71 (9.84) | t(1, 1716) = 1.20, *p* = .23 |
| IQ, mean (SD) | 100.95 (15.47) | 100.13 (14.92) | t(1, 485) =  0.59,  *p* = .56 | 109.73 (14.84) | 107.78 (15.11) | t(1, 227) =  0.98, *p* = .33 | n.d. | n.d. | n.d. |
| Inattentive scale, mean (SD)^a^ | 58.74 (13.89) | 57.69 (12.62) | t(1, 485) =  0.84,  *p* = .40 | 3.31 (3.26) | 4.09 (3.41) | t(1, 227) =  -1.76, *p* = .08 | 1.18 (1.66) | 1.23 (1.66) | t(1, 972) =  -0.42, *p* = .67 |
| Hyperactive/impulsive scale, mean (SD)^a^ | 60.99 (16.58) | 60.10 (16.02) | t(1, 485) =  0.59, *p* = .56 | 2.96 (2.75) | 3.66 (3.19) | t(1, 227) =  -1.77, *p* = .08 | 1.73 (1.69) | 1.47 (1.58) | t(1, 972) =  2.38, *p* = .02 |
| Total brain volume in ml, mean (SD)^b^ | 1251.62 (123.39) | 1273.74 (123.39) | t(1, 485) =  -1.93, *p* = .79 | 1239.32 (111.98) | 1254.89 (117.86) | t(1, 227) =  -1.01, *p* = .31 | 1243.85 (121.05) | 1233.38 (119.25) | t(1, 1716) = 1.78, *p* = .07 |

^a^ For NeuroIMAGE cohort: measured with the Conners’ Parent Rating Scale–Revised (Conners et al. 1998). Values refer to *t* scores on the *DSM* Total, Inattentive Behavior, and Hyperactive-Impulsive Behavior scales (scales N, L, and M). For IMpACT and BIG cohorts: measured with the ADHD-DSM-IV Self Rating scale (Kooij et al., 2005).

^b^ Total brain volume is defined as the sum of total gray and white matter.

n.d. = not determined
