# Supplementary material for: Enlarged striatal volume in adults with ADHD carrying the 9-6 haplotype of the dopamine transporter gene DAT1
Source: J Neural Transm (Vienna). 2016 Mar 2;123:905–15. doi: 10.1007/s00702-016-1521-x (PMC4969340; doi:10.1007/s00702-016-1521-x)
Supplement: Supplementary file 2 — Supplementary material 2 (DOCX 22 kb) [file 702_2016_1521_MOESM2_ESM.docx]

Supplementary Table 2. Participant characteristics for the *DAT1* 10-6 carriers and non-carriers for the three cohorts included in this study.

|  | NeuroIMAGE(N = 487) | | | IMpACT (N = 229) | | | BIG (N = 1718) | | |
| --- | --- | --- | --- | --- | --- | --- | --- | --- | --- |
| Characteristics | *DAT1* 10-6 carriers  (N = 456) | *DAT1* 10-6 non-carriers  (N = 31) | Test of significance | *DAT1* 10-6 carriers  (N = 209) | *DAT1* 10-6 non-carriers  (N = 20) | Test of significance | *DAT1 10-6* carriers  (N = 1575) | *DAT1 10-6* non-carriers (N = 143) | Test of significance |
| Male, N (%) | 283 (62) | 18 (58) | χ^2^ = 0.20, *p* = .66 | 87 (42) | 5 (25) | χ^2^ = 0.96, *p* = .34 | 689 (44) | 60 (42) | χ^2^ = 0.17, *p* = .68 |
| Age in years, mean (SD) | 16.95 (2.22) | 16.95 (3.26) | t(1, 485) = 0.01, *p* = .99 | 36.69 (11.35) | 34.15 (11.43) | t(1, 227) = 0.72, *p* = .47 | 26.15 (10.65) | 25.15 (10.42) | t(1, 1716) = 1.07, *p* = .29 |
| IQ, mean (SD) | 100.68 (15.31) | 99.68 (14.36) | t(1, 485) = 0.35, *p* = .72 | 108.66 (15.12) | 111.34 (13.26) | t(1, 227) =  -0.77, *p* = .45 | n.d. | n.d. | n.d. |
| Inattentive scale, mean (SD)^a^ | 58.39 (13.52) | 57.23 (11.37) | t(1, 485) = 0.46, *p* = .65 | 3.60 (3.33) | 4.15 (3.54) | t(1, 227) =  -0.70, *p* = .48 | 1.18 (1.65) | 1.42 (1.74) | t(1, 972) =  -1.28, *p* = .20 |
| Hyperactive/impulsive scale, mean (SD)^a^ | 60.93 (16.48) | 56.18 (13.67) | t(1, 485) = 1.55, *p* = .12 | 3.27 (3.27) | 3.15 (3.13) | t(1, 227) = 0.17, *p* = .86 | 1.62 (1.67) | 1.58 (1.43) | t(1, 972) = 0.25, *p* = .80 |
| Total brain volume in ml, mean (SD)^b^ | 1260.73 (112.81) | 1260.66 (125.34) | t(1, 485) = 0.01, *p* = .99 | 1247.52 (112.20) | 1254.89 (147.74) | t(1, 227) =  -0.27, *p* = .79 | 1247.78 (128.22) | 1237.00 (119.34) | t(1, 972) = 1.03, *p* = .30 |

^a^ For NeuroIMAGE cohort: measured with the Conners’ Parent Rating Scale–Revised (Conners et al. 1998). Values refer to *t* scores on the *DSM* Total, Inattentive Behavior, and Hyperactive-Impulsive Behavior scales (scales N, L, and M). For IMpACT and BIG cohorts: measured with the ADHD-DSM-IV Self Rating scale (Kooij et al., 2005).

^b^ Total brain volume is defined as the sum of total gray and white matter.

n.d. = not determined
