# Supplementary material for: Enlarged striatal volume in adults with ADHD carrying the 9-6 haplotype of the dopamine transporter gene DAT1
Source: J Neural Transm (Vienna). 2016 Mar 2;123:905–15. doi: 10.1007/s00702-016-1521-x (PMC4969340; doi:10.1007/s00702-016-1521-x)
Supplement: Supplementary file 3 — Supplementary material 3 (DOCX 20 kb) [file 702_2016_1521_MOESM3_ESM.docx]

Supplementary Table 3. Participant characteristics for the *DAT1* 9-6 carriers and non-carriers for the three cohorts included in this study.

|  | NeuroIMAGE (N = 487) | | | IMpACT (N = 229) | | | BIG (N = 1718) | | |
| --- | --- | --- | --- | --- | --- | --- | --- | --- | --- |
| Characteristics | *DAT1* 9-6  carriers  (N = 73) | *DAT1* 9-6 non-carriers  (N = 414) | Test of significance | *DAT1* 9-6 carriers  (N = 38) | *DAT1* 9-6 non-carriers  (N = 191) | Test of significance | *DAT1* 9-6 carriers  (N = 249) | *DAT1* 9-6 non-carriers  (N = 1469) | Test of significance |
| Male, N (%) | 50 (68) | 251 (61) | χ^2^ = 1.62, *p* = .20 | 9 (24) | 83 (43) | χ^2^ = 5.16, *p* = .02 | 101 (41) | 648 (44) | χ^2^ = 1.09, *p* = .30 |
| Age in years, mean (SD) | 17.12 (2.88) | 16.93 (3.26) | t(1, 485) =  0.47, *p* = .64 | 37.66 (13.57) | 36.23 (10.89) | t(1, 227) = 1.02, *p* = .30 | 25.69 (10.42) | 26.13 (10.67) | t(1, 1716) =  -0.61, *p* = .55 |
| IQ, mean (SD) | 98.13 (14.39) | 101.05 (15.36) | t(1, 485) =  -1.50, *p* = .13 | 111.19 (15.32) | 108.43 (14.88) | t(1, 227) = 0.98, *p* = .33 | n.d. | n.d. | n.d. |
| Inattentive scale, mean (SD)^a^ | 58.11 (12.08) | 58.13 (13.62) | t(1, 485) =  -0.14, *p* = .89 | 4.92 (3.40) | 3.39 (3.28) | t(1, 227) = 2.61, *p* = .01 | 1.10 (1.46) | 1.22 (1.69) | t(1, 972) =  -0.78, *p* = .44 |
| Hyperactive/impulsive scale, mean (SD)^a^ | 59.84 (14.79) | 60.77 (16.62) | t(1, 485) =  -0.45, *p* = .65 | 4.42 (3.19) | 3.03 (2.87) | t(1, 227) = 2.68, *p* = .008 | 1.35 (1.58) | 1.66 (1.66) | t(1, 972) =  -2.07, *p* = .04 |
| Total brain volume in ml, mean (SD)^b^ | 1270.32 (119.62) | 1258.96 (125.33) | t(1, 485) =  0.72, *p* = .47 | 1226,00 (101.37) | 1252.57 (117.69) | t(1, 227) =  -1.22, *p* = .20 | 1237.01 (119.60) | 1238.05 (120.23) | t(1, 1716) =  -2.07, *p* = .90 |

^a^ For NeuroIMAGE cohort: measured with the Conners’ Parent Rating Scale–Revised (Conners et al. 1998). Values refer to *t* scores on the *DSM* Total, Inattentive Behavior, and Hyperactive-Impulsive Behavior scales (scales N, L, and M). For IMpACT and BIG cohorts: measured with the ADHD-DSM-IV Self Rating scale (Kooij et al., 2005).

^b^ Total brain volume is defined as the sum of total gray and white matter.

n.d. = not determined
