# Supplementary material for: Enlarged striatal volume in adults with ADHD carrying the 9-6 haplotype of the dopamine transporter gene DAT1
Source: J Neural Transm (Vienna). 2016 Mar 2;123:905–15. doi: 10.1007/s00702-016-1521-x (PMC4969340; doi:10.1007/s00702-016-1521-x)
Supplement: Supplementary file 4 — Supplementary material 4 (DOCX 17 kb) [file 702_2016_1521_MOESM4_ESM.docx]

Supplementary Table 4. Striatal volumes for *DAT1* 10/10 carriers and non-carriers for ADHD patients and controls separately from the IMpACT cohort.

|  | IMpACT ADHD (N = 118) | | |
| --- | --- | --- | --- |
|  | *DAT1 10/10* carriers (N = 61) | *DAT1 10/10* non-carriers (N = 57) | Regression of binary genotypes on individual striatal volumes^b^ |
|  | Mean (SE)^a^ | Mean (SE)^a^ | β (95% CI), *p-*value |
| Total striatum | 18.55 (0.17) | 19.19 (0.18) | -0.64; (-1.14;-0.14), .013 |
|  |  |  |  |
|  | IMpACT Controls (N = 111) | | |
|  | *DAT1 10/10* carriers (N = 69) | *DAT1 10/10* non-carriers (N = 42) | Regression of binary genotypes on individual striatal volumes^b^ |
|  | Mean (SE)^a^ | Mean (SE)^a^ | β (95% CI), *p-*value |
| Total striatum | 19.10 (0.16) | 18.73 (0.21) | 0.36 (-0.17;.89), .18 |

^a^ Means are based on estimated marginal means corrected for age, gender, and total brain volume.

^b^ For main effects, β (unstandardized regression coefficient) is equal to the difference in mean brain volumes (in ml) between the genotype groups adjusted for covariates in the model. Included covariates were diagnostic status, age, gender and, total brain volume.
