# Supplementary material for: Enlarged striatal volume in adults with ADHD carrying the 9-6 haplotype of the dopamine transporter gene DAT1
Source: J Neural Transm (Vienna). 2016 Mar 2;123:905–15. doi: 10.1007/s00702-016-1521-x (PMC4969340; doi:10.1007/s00702-016-1521-x)
Supplement: Supplementary file 6 — Supplementary material 6 (DOCX 19 kb) [file 702_2016_1521_MOESM6_ESM.docx]

Supplementary Table 6. Participant characteristics for ADHD and control subjects from the NeuroIMAGE cohort, matched for gender and age.

|  | NeuroIMAGE (N = 487) | | |
| --- | --- | --- | --- |
| Characteristics | ADHD  (N = 301) | Controls  (N = 186) | Test of significance |
| 10/10 carriers, N (%) | 117 (53) | 104 (47) | χ^2^ = 1.96, *p* = .16 |
| 10-6 carriers, N (%) | 171 (50) | 171 (50) | χ^2^ = 0.0, *p* = 1.00 |
| 9-6 carriers, N (%) | 26 (53) | 23 (13) | χ^2^ = 0.21, *p* = .65 |
| Male, N (%) | 81 (57) | 107 (48) | χ^2^ = 0.0, *p* = 1.00 |
| Age in years, mean (SD) | 17.14 (2.23) | 16.56 (3.04) | t(1, 485) = 1.75, *p* = .08 |
| IQ, mean (SD) | 97.03 (14.24) | 106.30 (13.53) | t(1, 485) = -6.33, *p* < .001 |
| Inattentive scale, mean (SD)^a^ | 66.89 (11.69) | 46.31 (5.75) | t(1, 485) = 21.21, *p* < .001 |
| Hyperactive/impulsive scale, mean (SD)^a^ | 70.97 (14.80) | 46.33 (5.05) | t(1, 485) = 21.17, *p* < .001 |
| Total brain volume in ml, mean (SD)^b^ | 1240.73 (125.19) | 1262.99 (123.13) | t(1, 485) = -1.73, *p* = .09 |

^a^ Measured with the Conners’ Parent Rating Scale–Revised (Conners et al. 1998). Values refer to *t* scores on the *DSM* Total, Inattentive Behavior, and Hyperactive-Impulsive Behavior scales (scales N, L, and M).

^b^ Total brain volume is defined as the sum of total gray and white matter.
