# Supplementary material for: Enlarged striatal volume in adults with ADHD carrying the 9-6 haplotype of the dopamine transporter gene DAT1
Source: J Neural Transm (Vienna). 2016 Mar 2;123:905–15. doi: 10.1007/s00702-016-1521-x (PMC4969340; doi:10.1007/s00702-016-1521-x)
Supplement: Supplementary file 7 — Supplementary material 7 (DOCX 18 kb) [file 702_2016_1521_MOESM7_ESM.docx]

Supplementary Table 7. Striatal volumes and regression analyses testing differences between *DAT1* 10/10, 10-6, 9-6 carriers and non-carriers in a subsample of the NeuroIMAGE cohort, matched for gender and age.

|  | NeuroIMAGE (N = 362) | | |
| --- | --- | --- | --- |
|  | *DAT1* 10/10 carriers (N = 221) | *DAT1* 10/10 non-carriers (N = 141) | Regression of binary genotypes on individual striatal volumes^b^ |
|  | Mean (SE)^a^ | Mean (SE)^a^ | β (95% CI), *p-*value |
| Total striatum | 20.05 (0.09) | 19.92 (0.12) | 0.13; (-0.17;0.43), .39 |
|  |  |  |  |
|  | *DAT1* 10-6 carriers (N = 342) | *DAT1* 10-6 non-carriers (N = 20) | Regression of binary genotypes on individual striatal volumes^b^ |
|  | Mean (SE)^a^ | Mean (SE)^a^ | β (95% CI), *p-*value |
| Total striatum | 19.97 (0.08) | 20.51 (0.21) | -0.53 (-1.16;.10), .098 |
|  |  |  |  |
|  | *DAT1* 9-6 carriers (N = 49) | *DAT1* 9-6 non-carriers (N = 313) | Regression of binary genotypes on individual striatal volumes^b^ |
|  | Mean (SE)^a^ | Mean (SE)^a^ | β (95% CI), *p-*value |
| Total striatum | 19.81 (0.20) | 20.03 (0.79) | -0.23 (-0.65;.19), .29 |

^a^ Means are based on estimated marginal means corrected for age, gender, and total brain volume.

^b^ For main effects, β (unstandardized regression coefficient) is equal to the difference in mean brain volumes (in ml) between the genotype groups adjusted for covariates in the model. Included covariates were diagnostic status, age, gender, and total brain volume.
