# Supplementary material for: Enlarged striatal volume in adults with ADHD carrying the 9-6 haplotype of the dopamine transporter gene DAT1
Source: J Neural Transm (Vienna). 2016 Mar 2;123:905–15. doi: 10.1007/s00702-016-1521-x (PMC4969340; doi:10.1007/s00702-016-1521-x)
Supplement: Supplementary file 8 — Supplementary material 8 (DOCX 20 kb) [file 702_2016_1521_MOESM8_ESM.docx]

Supplementary Table 8. Participant characteristics for the *DAT1* 10/10, *DAT1* 9-6 carriers, and non-carriers from the IMpACT cohort, matched for gender and age.

|  | IMpACT (N = 194) | | |
| --- | --- | --- | --- |
| Characteristics | *DAT1* 10/10 carriers (N = 97) | *DAT1* 10/10 non-carriers (N = 97) | Test of significance |
| Male, N (%) | 9 (26) | 69 (42) | χ^2^ = 1.12, *p* = .29 |
| Age in years, mean (SD) | 37.72 (12.18) | 35.64 (11.00) | t(1, 192) = 1.25, *p* = .21 |
| IQ, mean (SD) | 109.25 (14.67) | 107.61 (15.17) | t(1, 192) = 0.77, *p* = .45 |
| Inattentive scale, mean (SD)^a^ | 3.31 (3.35) | 4.04 (3.42) | t(1, 192) = -1.50, *p* = .73 |
| Hyperactive/impulsive scale, mean (SD)^a^ | 3.02 (2.76) | 3.58 (3.17) | t(1, 192) = -1.31, *p* = .19 |
| Total brain volume in ml, mean (SD)^b^ | 1237.59 (114.58) | 1237.04 (111.92) | t(1, 192) = 0.34, *p* = .97 |
|  |  |  |  |
|  | IMpACT (N = 200) | | |
| Characteristics | *DAT1* 9-6 carriers (N = 34) | *DAT1* 9-6 non-carriers (N = 166) | Test of significance |
| Male, N (%) | 9 (26) | 69 (42) | χ^2^ = 1.12, *p* = .29 |
| Age in years, mean (SD) | 38.00 (13.26) | 35.59 (10.77) | t(1, 198) = 1.14, *p* = .26 |
| IQ, mean (SD) | 110.65 (15.00) | 109.50 (14.94) | t(1, 198) = 0.41, *p* = .68 |
| Inattentive scale, mean (SD)^a^ | 4.76 (3.55) | 3.28 (3.27) | t(1, 198) = 2.37, *p* = .02 |
| Hyperactive/impulsive scale, mean (SD)^a^ | 4.12 (3.22) | 2.98 (2.90) | t(1, 198) = 2.05, *p* = .04 |
| Total brain volume in ml, mean (SD)^b^ | 1217.18 (99.67) | 1252.57 (110.48) | t(1, 198) = -1.73, *p* = .09 |

^a^ Measured with the ADHD-DSM-IV Self Rating scale (Kooij et al., 2005).

^b^ Total brain volume is defined as the sum of total gray and white matter.
