# Supplementary material for: Enlarged striatal volume in adults with ADHD carrying the 9-6 haplotype of the dopamine transporter gene DAT1
Source: J Neural Transm (Vienna). 2016 Mar 2;123:905–15. doi: 10.1007/s00702-016-1521-x (PMC4969340; doi:10.1007/s00702-016-1521-x)
Supplement: Supplementary file 10 — Supplementary material 10 (DOCX 17 kb) [file 702_2016_1521_MOESM10_ESM.docx]

Supplementary Table 10. Striatal volumes and regression analyses testing differences between *DAT1* 9-6 carriers and non-carriers in the IMpACT cohort.

|  | IMpACT (N = 229) | | |
| --- | --- | --- | --- |
|  | *DAT1* 9-6 carriers (N = 38) | *DAT1* 9-6 non-carriers (N = 191) | Regression of binary genotypes on individual striatal volumes^b^ |
|  | Mean (SE)^a^ | Mean (SE)^a^ | β (95% CI), *p-*value |
| Left accumbens | 0.61 (0.015) | 0.61 (0.007) | 0.01 (0.03;0.04), .84 |
| Right accumbens | 0.55 (0.013) | 0.52 (0.006) | 0.03 (0.01;0.06), .04 |
| Left caudate | 3.81 (0.059) | 3.61 (0.026) | 0.20 (0.07;0.33), .002 |
| Right caudate | 3.92 (0.059) | 3.70 (0.026) | 0.22 (0.09;0.35), .001 |
| Left putamen | 5.52 (0.80) | 5.20 (0.035) | 0.32 (0.14;0.49), .0004 |
| Right putamen | 5.42 (0.072) | 5.10 (0.032) | 0.33 (0.17;0.48), .00005 |

^a^ Means are based on estimated marginal means corrected for diagnosis, age, gender, and total brain volume; for the NeuroIMAGE and BIG cohorts, covariates also included scanner type/location; for the BIG cohort, diagnostic status was dropped from the model.

^b^ For main effects, β (unstandardized regression coefficient) is equal to the difference in mean brain volumes (in ml) between the genotype groups adjusted for covariates in the model. Included covariates were age, gender, and total brain volume.
