# Supplementary material for: Enlarged striatal volume in adults with ADHD carrying the 9-6 haplotype of the dopamine transporter gene DAT1
Source: J Neural Transm (Vienna). 2016 Mar 2;123:905–15. doi: 10.1007/s00702-016-1521-x (PMC4969340; doi:10.1007/s00702-016-1521-x)
Supplement: Supplementary file 11 — Supplementary material 11 (DOCX 18 kb) [file 702_2016_1521_MOESM11_ESM.docx]

Supplementary Table 11. Striatal volumes and regression analyses testing differences between *DAT1* 9-6 carriers and non-carriers for ADHD patients and controls separately from the IMpACT cohort.

|  | IMpACT ADHD (N = 118) | | |
| --- | --- | --- | --- |
|  | *DAT1* 9-6 carriers (N = 26) | *DAT1* 9-6 non-carriers (N = 92) | Regression of binary genotypes on individual striatal volumes^b^ |
|  | Mean (SE)^a^ | Mean (SE)^a^ | β (95% CI), *p-*value |
| Left accumbens | 0.64 (0.015) | 0.61 (0.007) | 0.03 (-0.01;0.06), .13 |
| Right accumbens | 0.54 (0.015) | 0.51 (0.008) | 0.04 (0.01;0.07), .05 |
| Left caudate | 3.83 (0.073) | 3.60 (0.038) | 0.24 (0.08;0.40), .005 |
| Right caudate | 3.94 (0.073) | 3.67 (0.038) | 0.26 (0.10;0.43), .002 |
| Left putamen | 5.56 (0.100) | 5.14 (0.052) | 0.42 (0.20;0.64), .0003 |
| Right putamen | 5.41 (0.087) | 5.03 (0.045) | 0.39 (0.19;0.58), .0001 |
|  | | | |
|  | IMpACT Controls (N = 111) | | |
|  | *DAT1* 9-6 carriers (N = 26) | *DAT1* 9-6 non carriers (N = 85) | Regression of binary genotypes on individual striatal volumes^b^ |
|  | Mean (SE)^a^ | Mean (SE)^a^ | β (95% CI), *p-*value |
| Left accumbens | 0.57 (0.015) | 0.60 (0.007) | 0.03 (-0.10;0.03), .26 |
| Right accumbens | 0.55 (0.023) | 0.53 (0.008) | -0.03 (-0.03;0.08), .30 |
| Left caudate | 3.76 (0.101) | 3.62 (0.035) | 0.14 (-0.07;0.35), .19 |
| Right caudate | 3.87 (0.104) | 3.72 (0.036) | 0.15 (-0.07;0.37), .18 |
| Left putamen | 5.36 (0.135) | 5.26 (0.047) | 0.10 (-0.18;0.39), .49 |
| Right putamen | 5.36 (0.127) | 5.17 (0.044) | 0.19 (-0.08;0.46), .16 |

^a^ Means are based on estimated marginal means corrected for age, gender, and total brain volume.

^b^ For main effects, β (unstandardized regression coefficient) is equal to the difference in mean brain volumes (in ml) between the genotype groups adjusted for covariates in the model. Included covariates were age, gender, and total brain volume.
