# Supplementary material for: Enlarged striatal volume in adults with ADHD carrying the 9-6 haplotype of the dopamine transporter gene DAT1
Source: J Neural Transm (Vienna). 2016 Mar 2;123:905–15. doi: 10.1007/s00702-016-1521-x (PMC4969340; doi:10.1007/s00702-016-1521-x)
Supplement: Supplementary file 12 — Supplementary material 12 (DOCX 16 kb) [file 702_2016_1521_MOESM12_ESM.docx]

Supplementary Table 12. Regression of binary genotypes on total striatal volume corrected for lifetime medication use^a^

|  | NeuroIMAGE (N = 487) | IMpACT (N = 229) |
| --- | --- | --- |
|  | β (95% CI), *p-*value^b^ | β (95% CI), *p-*value^b^ |
| 10/10 | 0.21 (-0.06;0.48), .13 | -0.17 (-0.53;0.20), .37 |
| 10-6 | -0.40 (-0.70;0.38), .15 | -0.41 (-1.05;0.22), .20 |
| 9-6 | -0.27 (-0.63;0.09), .14 | 1.10 (0.63;1.56), .00001^c^ |

^a^ Results from the final regression model examining associations between binary genotype (risk carriers vs non-risk carriers) and brain volumes.

^b^ For main effects, β (unstandardized regression coefficient) is equal to the difference in mean brain volumes (in ml) between the genotype groups adjusted for covariates in the model. Included covariates were diagnosis, age, gender, total brain volume and lifetime medication use; for the NeuroIMAGE sample, covariates also included scanner type.

^c^ β = 1.10 denotes that 9-6 carriers had a 1.10 ml larger striatum volume than non 9-6 carriers.
